# E-Cigarette Social Media Messages: A Text Mining Analysis of Marketing and Consumer Conversations on Twitter

**DOI:** 10.2196/publichealth.6551

**Published:** 2016-12-12

**Authors:** Allison J Lazard, Adam J Saffer, Gary B Wilcox, Arnold DongWoo Chung, Michael S Mackert, Jay M Bernhardt

**Affiliations:** ^1^School of Media and JournalismUniversity of North Carolina at Chapel HillChapel Hill, NCUnited States; ^2^Center for Health CommunicationMoody College of CommunicationThe University of Texas at AustinAustin, TXUnited States; ^3^Center for Health CommunicationStan Richards School of Advertising and Public RelationsThe University of Texas at AustinAustin, TXUnited States

**Keywords:** e-cigarettes, social media, tweet, Internet

## Abstract

**Background:**

As the use of electronic cigarettes (e-cigarettes) rises, social media likely influences public awareness and perception of this emerging tobacco product.

**Objective:**

This study examined the public conversation on Twitter to determine overarching themes and insights for trending topics from commercial and consumer users.

**Methods:**

Text mining uncovered key patterns and important topics for e-cigarettes on Twitter. SAS Text Miner 12.1 software (SAS Institute Inc) was used for descriptive text mining to reveal the primary topics from tweets collected from March 24, 2015, to July 3, 2015, using a Python script in conjunction with Twitter’s streaming application programming interface. A total of 18 keywords related to e-cigarettes were used and resulted in a total of 872,544 tweets that were sorted into overarching themes through a text topic node for tweets (126,127) and retweets (114,451) that represented more than 1% of the conversation.

**Results:**

While some of the final themes were marketing-focused, many topics represented diverse proponent and user conversations that included discussion of policies, personal experiences, and the differentiation of e-cigarettes from traditional tobacco, often by pointing to the lack of evidence for the harm or risks of e-cigarettes or taking the position that e-cigarettes should be promoted as smoking cessation devices.

**Conclusions:**

These findings reveal that unique, large-scale public conversations are occurring on Twitter alongside e-cigarette advertising and promotion. Proponents and users are turning to social media to share knowledge, experience, and questions about e-cigarette use. Future research should focus on these unique conversations to understand how they influence attitudes towards and use of e-cigarettes.

## Introduction

Since the introduction of electronic nicotine delivery systems or electronic cigarettes (e-cigarettes) less than a decade ago, awareness and use of these products has risen dramatically [1-5]. E-cigarettes are battery-powered devices that deliver varying amounts of nicotine, propylene glycol, and flavorings, among other things, through an aerosolized liquid solution (commonly referred to as vaping) [6]. Introduced as a substitution for cigarettes, e-cigarette use has grown rapidly despite little knowledge about the short- and long-term health risks or the broad impact on public health given high levels of dual use of e-cigarettes and conventional cigarettes [6-9]. Concerns for increased e-cigarette use are not limited to dual-users. Lifetime or ever use of e-cigarettes has increased exponentially among adults, and more importantly, those most susceptible to addiction, adolescents and young adults [10-13].

As the prevalence of e-cigarettes increases, researchers must understand how individuals acquire knowledge about these products and consider how the different sources of information might influence decisions of use. Prior research has consistently found that individuals are exposed to, search for, and share information about e-cigarettes on social media platforms like Twitter and Facebook, among others [14-17]. Interestingly, e-cigarette users are “more likely to be exposed to e-cigarette information via TV viewing sites (eg, Hulu), Twitter, Facebook, email, and Internet search engines” than nonusers [15]. Therefore, it is important to understand what information e-cigarette users and potential users are exposed to on social media.

Twitter is particularly unique among social media platforms because users can broadcast their messages in the form of tweets and retweets to large audiences [18-20], often sharing information that may contribute to conceptualizations of new health products and phenomena [17,21,22]. Broadly speaking, there are 3 types of users tweeting about e-cigarettes: individual users representative of the public, e-cigarette proponents, and commercial marketers. Individual users are average people who tweet or retweet messages about e-cigarettes that can directly reach their followers or indirectly reach other users following hashtags or key terms in a tweet. Proponents are a diverse group of representatives of e-cigarette organizations, vaping advocates, and those who identify as e-cigarette users in their online profiles [23]. Notably, proponents have been shown to tweet e-cigarette content 5 times as often as individual users, much of which is positive toward e-cigarette use [23]. Commercial marketers, such as e-cigarette retailers and manufacturers, are those who use Twitter to promote and advertise their products. Individuals can be exposed to information about e-cigarettes from individual, proponent, or commercial sources.

Many studies have found that exposure to e-cigarette messages on Twitter has increased significantly in recent years [24-26]. As of 2016, 1 study found tweets about e-cigarettes have increased 5 times since 2012 [26]. Further investigations into the sources of e-cigarette tweets indicate that most tweets originate from commercial users as marketing or advertising promotional messages. According to Huang and colleagues [27], 90% of e-cigarette tweets are from commercial users, and Kim et al [24] reported that 93.43% of tweets were for advertising.

However, the data collected for these studies may have had some confounding variables. Huang et al [27] noted that “the time frame during which our data were collected was just prior to the launch of major e-cigarette TV marketing.” Kim et al [24] analyzed data from the same time frame. This may have led to the finding that most e-cigarette tweets come from commercial users. Some evidence is emerging that challenges previously reported prevalence of e-cigarette tweets from commercial users and for advertising. Dai and Hao [28], who analyzed individual users’ organic tweets about e-cigarettes, found that 10.8% supported, 17.7% were against, and 19.4% were neutral toward the use of e-cigarettes [28], indicating there is variance in the public conversation about e-cigarettes on Twitter.

An additional confound is the types of users tweeting about e-cigarettes. To understand individual exposures, it is necessary to reveal the type of accounts generating e-cigarette–related tweets. Researchers must first recognize the prevalence of automated social bot accounts (also referred to as cyborgs) run by third parties to influence and promote e-cigarettes and related products but resembling average users [29,30]. Tweets from bot accounts are reducing the signal-to-noise ratio where individuals’ e-cigarette tweets are far fewer.

For instance, Clark et al [31] looked at a 10% sample of e-cigarette tweets from “Twitter’s garden hose” over a 2-year period and found that 80% of tweets were “automated and promotional.” Thus, it is necessary to increase the signal-to-noise ratio to accurately examine e-cigarette–related Twitter discussions among individuals, and denoising techniques allow researchers to do so [29]. However, Allem and Ferrara [29] cautioned researchers against using “crude” and “blunt” techniques such as removing tweets with links solely relying on community detection or methods solely relying on innocent-by-association paradigms. While denoising techniques are still emerging, this work highlights an important need for awareness of the signal-to-noise issue for surveillance of social media data.

Searching for the signal, themes of e-cigarette tweets have been studied to reveal salient topics and sentiment of publically accessible conversations on Twitter. Using a priori themes, Kavuluru and Sabbir [23] detected largely positive sentiment for themes of flavors, harm reduction, smoke-free aspects, and smoking cessation. Similarly and on a small scale relative to the current data-mining abilities, Myslin and colleagues [32] used a combination of a priori themes and iterative machine learning to identify a preponderance of first- or second-hand experience tweets about tobacco-related themes of hookah, cessation, and pleasure. E-cigarette content was not among the top themes, likely given the newness of these products in 2011-2012; however, a trend of positive sentiment with e-cigarette content was detected (contrary to more negative sentiment with smoking tweets) [32]. Cole-Lewis and colleagues [33,34], also using themes developed from previous research, revealed that advertising/promotion, policy/government, and health/safety are among the most dominant themes. Much of this content had a positive sentiment skewing favorably toward e-cigarettes [33]. In a purposeful sample specific for smoking cessation, van der Tempel and colleagues [26] found similar dominant themes—marketing, news, personal experiences—from an a priori theme list. Together, the trends in the literature indicate that as e-cigarette content becomes more popular, the majority of this content is positive promotion. However, missing still is large-scale, inductive analysis of the topics and themes of the tweets from all categories of users. Our study presents the topics and themes of tweets about e-cigarettes from individual, proponent, and commercial users.

An accurate understanding of the types of users tweeting, as well as what they are tweeting, about e-cigarettes can provide a better understanding of what individuals are being exposed to on social media. Looking specifically at the nonadvertising tweets in their data, Kim et al [24] found that organic conversations are occurring online among individuals about e-cigarettes. This is particularly important because organic conversations may affect individual exposure. That is, if individuals are exposed to an e-cigarette tweet from another individual they know, they may be more easily persuaded given the relational closeness and potentially stronger levels of source credibility [35,36]. Thus, as the use of e-cigarettes continues to rise, public awareness of these products is likely shaped by the proliferation of messages shared and reshared on social media. To understand this media landscape, this study used a textual analysis method to examine the public conversation on Twitter and determine overarching themes and trending topics from commercial and consumer contributors.

## Methods

### Text Mining and Data Acquisition

This study used a text-mining approach to uncover key patterns and relationships within unstructured data to understand and evaluate information important to the audience. Text mining is the term used to describe either a single process or a collection of processes in which software tools actively engage in the “discovery of new, previously unknown information by automatically extracting information from different written (or text) sources” [37]. Text mining provides an opportunity to uncover key patterns and relationships within both structured and unstructured data and allows researchers to more easily understand and evaluate information important to the audience.

In the area of public health, text mining of social media has been used to detect and track disease outbreaks and estimate the level of public knowledge regarding health issues [38]. Twitter was selected for data collection due to its popularity as a microblog as well as the active nature of its users in sending messages to create conversations regarding the use of new products and related social issues [18,21]. The central challenge of text mining is the analysis of unstructured data in order to extract meaningful associations, trends, and patterns in large amounts of text. The increasing availability and magnitude of unstructured digital data available in social media such as Twitter, Facebook, blogs, and other online environments offers new opportunities for researchers to investigate social, cultural, and health issues.

The methodology and workflow for this study depended upon a combination of human and technological analysis of the Twitter messages and employed 5 steps. First, the search term and time period were determined. Next, Twitter’s streaming application programming interface (API) was used to acquire the data. Third, researchers cleaned the data and removed duplication and unrecognized characters. Fourth, data were processed using text-mining software and fifth, the findings were interpreted.

### Data Collection

Twitter was selected for data collection due to its popularity as a microblogging service and the active nature of its users in sending messages regarding news and social issues, including health-related issues. Tweets were collected from March 24, 2015, to July 3, 2015, using a Python script in conjunction with Twitter’s streaming API. A total of 18 keywords related to e-cigarettes, vaping, and e-cigarette brands were used (ecigs, ecigarettes, e-cigarettes, electronic cigarettes, vaping, vapestick, ehookah, ejuice, Blu eCigs, E-Swisher, Ezsmoker, Fin, NJOY/NJOY, Smoke Assist, V2 Cigs, MarkTen, Vuse, and Tryst) which resulted in a total of 872,544 tweets and retweets. Tweets and retweets were separated into 2 files for the analysis, a tweet file containing 546,651 entries and a retweet file containing 325,893 entries.

### Data Analysis

Analysis of the textual content of the tweets was conducted using SAS Text Miner version 12.1 (SAS Institute Inc). SAS Text Miner allowed the researchers to parse and extract information from text, filter, and assemble documents into related topics allowing the researchers to discover topics and understand the data. This software was used for descriptive text-mining purposes to uncover the primary topics that were being discussed during the 100 days examined.

Following the collection of the data, the initial step was to extract, clean, and create a dictionary of words from the data using a natural language processor. A node process flow was created in SAS Enterprise Miner Workstation version 12.1 (SAS Institute Inc). It began with a Text Parsing node where each tweet is divided into tokens (terms). Specifically, this includes identifying sentences, determining parts of speech, and stemming words. Words were spell-checked and parsed to identify entities and remove stop words. The identified tokens or terms were listed in a “term by frequency” matrix via a text transformation of the numerical representation of the text using linear algebra–based priority models. To ensure that words that contribute little were not overly emphasized by the algorithm, the following parts of speech were ignored: auxiliary verbs, conjunctions, determiners, interjections, participles, prepositions, and pronouns.

Next in the Text Filter node, terms that appeared in fewer than 10 messages were ignored. The data were filtered using Entropy as the term weight and Log as the frequency weighting. The term filtering alters the term-by-document matrix, which contains the frequency of the occurrence of the term in the documents as the value of each cell. From this frequency matrix, a weighted term-by-document matrix was generated using software-driven term-weighting techniques. Within the Text Filter, the Filter Viewer was employed to visually inspect the individual terms. Unrecognizable symbols and letter groupings were manually excluded. Next, a check was made of the terms that were ignored to ascertain if any should be included in the analysis. A single author who had knowledge of the subject matter manually excluded irrelevant terms. Finally, the Text Filter node was used to reduce the total number of parsed terms, thereby eliminating extraneous information and retaining the most relevant parts of the text.

The Text Topic node was then employed to combine terms into topic groups. SAS Text Miner uses 2 types of clustering algorithms: expectation maximization (EM) and hierarchical clustering. EM clustering was used because it allows for and automatically selects between 2 versions of the algorithm—1 for small data files (standard) and 1 for larger (scaled) data files. Since there were over 800,000 tweets and retweets, the EM option was preferred. Options were selected within the software to create topic groups to include all topics that contained more than 1% of the total tweets or retweets. Topics with less than 5467 tweets or 3259 retweets were excluded from the analysis given they represented less than 1% of the data.

Last, the Topic Viewer option in Text Topic was used to further refine and interpret the topic groups. Individual tweets and retweets were reviewed and used to create summaries of each topic group. One author evaluated the results by completing several different iterations of SAS Text Miner, comparing the different results, and selecting what appeared to be the optimum solution after careful inspection of the output. After visual examination of each topic list (9 tweet topics and 14 retweet topics), topics that clearly did not illustrate the main themes were removed to reduce noise. Individual review of the actual topics generated by the software was undertaken to further exclude topics that appeared from automated accounts. This was accomplished by individually reviewing the actual messages from each topic to produce the final grouping of topics for tweets (8 topics) and retweets (5 topics) with the description in Tables 1 and 2. This process aided in noise reduction among the tweets as called for by Allem and Ferrar [29].

**Table 1:** E-cigarette tweets by topic.

	SAS Text Miner topic	n	Description of topic	Category
1	+E-cigarette, +smoke, ecigs, +quit, vaping	29,556	Pro-vaping discussion whether e-cigarettes are cessation devices or gateway products for youth to establish nicotine addictions	Proponent and individual user conversation
2	Ejuice, eliquid, vape, vapelife, ecig	24,064	Pro-vaping reactions to e-cigarette policies and questions about e-cigarette health risk claims	Proponent and individual user conversation
3	Vaping, vape, ecigs, vapelife, vapecommunity	21,555	News and updates from the vaping community, primarily through a vaping blog’s daily updates	Proponent and individual user conversation
4	Vapeporn, +tree, vape, vaping, reddit	16,414	Vaping advocacy encouraging the uptake of vaping and tips for users	Proponent and individual user conversation
5	Electronic, +cigarette, +electronic cigarette, +employee, +relieve	12,694	Employees may use e-cigarettes at work as relief from smoking bans	Proponent and individual user conversation
6	+Juice, vapejuice, +vaporizer, +vapor, vape	9092	Promotion for e-juice	Marketing/advertising
7	+Well price, +vapour, +price, +good,	6860	Price promotion for e-cigarettes	Marketing/advertising
8	+Win, cigbuyer, vapegiveaway, +enter, +sampler	5892	Promotion for an e-liquid give-away	Marketing/advertising

**Table 2:** E-cigarette retweets by topic.

	SAS Text Miner topic	n	Description of topic	Category
1	Vapinxsmoker, vaping, ecigs, +smoke, +e-cigarette	42,430	Discussion of policies banning e-cigarette use, e-cigarettes as (or not as) smoking cessation devices, and differentiation of e-cigarettes from traditional cigarettes	Proponent and individual user conversation
2	Vape, ejuice, ecigs, +win, vaping	33,767	Promotions for an e-juice give-away	Marketing/advertising
3	Amp, +win, +follow, +chance, http	18,305	Promotions for a chance to win an e-cigarette	Marketing/advertising
4	Vape, realdonnadevane, vaping, granny, ur	13,752	E-cigarette blogger suggests ways to “grow your #Vape business”	Marketing/advertising
5	+Starter, hookahcoals, http://t.co/5amkbtyrp3, newyork, eastcoast	6197	Promotions for e-hookah starter kits	Marketing/advertising

## Results

### Overview

Of the 872,544 tweets and retweets captured from March to July 2015, 240,578 were included in the final topic groups that each represented more than 1% of the conversation on Twitter during this time period for tweets and retweets. These were divided among 126,127 tweets sorted into 8 unique topics, shown in Table 1, and 114,451 retweets in 5 topics, shown in Table 2. Each table shows the topics featuring the prevalent keywords as generated by SAS Text Miner, number of tweets per topic, a description of the topic, and whether the topic represents proponent and individual user conversations or marketing/advertising. All topics with diverse tweets not directly linked to marketing were labeled proponent and individual user conversations. Topics dominated by promotions or branded persuasive appeals were categorized as marketing/advertising; often a single, repeated tweet comprised the entire topic for marketing/advertising.

### Tweet Topics

Of the 8 topics generated from the tweets, the top 5 topics, determined by number of tweets contained in the topic, included diverse proponent and individual user conversations. The most popular topic in the analysis represented a diverse public conversation that covered whether e-cigarettes are cessation devices or gateway products to get youth addicted to nicotine. This topic was dominated by pro–e-cigarette content and included comments that e-cigarettes may help people quit smoking, that the rise in e-cigarette use among adolescents may be deterring them from traditional tobacco experimentation, how e-cigarette bans may have unintended consequences, and claims and questions about whether the science showing the risk of e-cigarettes is flawed. Anti–e-cigarette tweets within this topic were limited but did include warnings for adolescent use.

The second most popular topic was a proponent and individual user conversation in reaction to e-cigarette bans and proposed taxes along with further questioning of whether there is evidence to support health risk claims about e-cigarette use. This topic represents a conversation around efforts to differentiate e-cigarettes from traditional tobacco products by pointing to the lack of evidence for the harm or risks of e-cigarettes along with the position that e-cigarettes should be promoted as smoking cessation devices, aside from the youth discussion above, by generally taking the stance that the use of e-cigarettes can save lives. Additionally, concerns expressed that products would be too expensive or unavailable because of regulation were also common.

The third topic from the tweets contained e-cigarette news and updates from proponents in the vaping community, although this community was dominated by one pro–e-cigarette news outlet. Many of these tweets were generated through the “share” option from the daily vaping news website and covered a range of topics that included coverage of policies (eg, bans, taxes), promotions from the organization, product reviews, tips for social and culture practices of vaping, and diverse articles that highlight e-cigarettes as cessation devices, along with the uncertainty of risk with e-cigarette use (eg, “Is a daily dose of nicotine as benign as coffee?”). This topic also included notices of new products available for sale from a variety of distributors.

The fourth topic consisted of vaping advocacy comments from a variety of proponent and individual user angles. Tweets included tips for e-cigarette users, discussions of flavors, encouragement for expanding the social practices of vaping (via new people and new places), and information about the use of specific devices as well as using devices for marijuana consumption. The fifth proponent and individual user topic consisted of a discussion about how employees may use e-cigarettes as a way to find relief from smoking bans at workplaces, as well as providing information about e-cigarettes and vaping.

Topics 6 through 8, the least populated topics, contained marketing promotions from 3 unique vendors. Each of these topics contained a single repeated tweet or tweets with only slight variations. Topic 6 contained just over 9000 original tweets that promoted a single distributor’s e-juice in a variety of flavors, such as kettle corn, grape, vanilla, and menthol. Topic 7 was entirely the repetition of one price promotion tweet from a different distributor, merely mentioning they had the best prices. Topic 8 was similarly the repeat of a single tweet; the tweet was a give-away promotion for an e-liquid sampler from a third distributor. The sampler promotion contained four 30 mL bottles in flavors *gravel pit*, *lime cola*, *strawberry blonde*, and *trail mix.*

### Retweet Topics

The 5 retweet topics consisted of 1 proponent and individual user conversation topic and 4 marketing/advertising topics. The most popular topic—the proponent and individual user conversation—contained references to policy bans for using e-cigarettes in public places and raising age restrictions for the legal purchase of tobacco products; comments about the likelihood that e-cigarette use does (or does not) lead to smoking cessation, with a heavy emphasis on how switching to e-cigarettes may not help users quit; and arguments for the differentiation between vaping and smoking, often framed as a pro-vaping argument focused on the reduced risk of e-cigarette use compared to smoking cigarettes.

The remaining topics, which make up over 60% of the retweeted content, were comprised exclusively of 4 unique messages retweeted over 70,000 times. Most (3 of the 4) retweeted messages were promotions to win a free bottle of e-juice, a variable wattage mod style e-cigarette, or an e-hookah starter kit. The remaining marketing/advertising topic of retweeted messages was a promotion for a single pro–e-cigarette blogger as an endorser and consultant for vape businesses.

## Discussion

### Principal Findings

As the use of e-cigarettes continues to rise, public awareness and perception of these products are likely shaped by the proliferation of messages shared and reshared on social media [26]. This study examined the public conversation on Twitter to determine overarching themes and trending topics. Topics found in this study included whether e-cigarettes are cessation devices or gateway products for tobacco addiction, how e-cigarettes differ from traditional cigarettes, reactions to e-cigarette policies and health risk claims, news and updates from vaping communities, use of e-cigarettes where smoking bans exist, and a variety of marketing product promotions and giveaways. While past research has found a preponderance of marketing and advertising dominating the content on Twitter [24,27], this study revealed that proponents and individual users are participating in public conversations about e-cigarettes at a much larger scale than previously suggested. However, the diversity in content did not reveal a conversation with diverse perspectives. Individual user and proponent tweets intertwined with marketing messages, which still have a strong presence, to present a rather unbalanced, likely proponent-driven and perpetuated conversation about e-cigarettes use, norms, and policy.

In contrast to earlier studies, this study is the first to indicate that public conversations, from a mix of individual users and proponents, are now dominating the trending topics on Twitter for e-cigarettes, even with the inclusion of commercial activity [26]. These topics, with over 800,000 tweets generated in a 100-day window, provide rich insights into salient issues for Twitter users, especially for those who support the use of e-cigarettes. Advocates for e-cigarettes have taken to Twitter to share their thoughts and opinions, contributing to an unbalanced, likely proponent-driven conversation about e-cigarettes that heavily favors pro–e-cigarette arguments. Notably, although the most popular topics were labeled proponent and individual users conversations, these were not free of industry influence via a mixture of marketing tweets within as well as messages clearly influenced by marketing strategies from users and industry members alike. Content from e-cigarette proponents reflects the indirect marketing influence in these conversations.

It is perhaps not surprising that those passionate enough to tweet are talking about the benefits of e-cigarette use. Similar trends of Twitter conversations dominated with pro–e-cigarette content have been detected in response to e-cigarette educational campaigns and announcements of e-cigarette regulations [39,40]. Our findings, which mirror these trends of countercampaigns and antipolicy Twitter bombing to flood conversations with one perspective [39,40], highlight the pressing need for public health professionals to engage the public on social media. This finding reveals perhaps an even larger concern for all public health professionals: each person who goes online to do a little research about e-cigarettes is going to encounter a tilted conversation encouraging e-cigarette use, promoting vaping as a socially acceptable practice for all ages, discrediting scientific evidence for health risks, and rallying around the idea that e-cigarettes should largely be outside the bounds of policy. Thinking about how public health advocates can either more actively engage in this conversation or encourage a broader range of the public, inclusive of those with neutral or anti–e-cigarette positions, to post is necessary to create a more balanced conversation.

Marketing and advertising still have a strong presence on Twitter; however, our results only partially support what others have found. Previous studies have suggested that marketing content saturates over 90% of the information about e-cigarettes on Twitter [24,27]. Although still present in this sample, this analysis revealed a much smaller proportion of marketing/advertising content as the most popular topics, especially for tweets. The fact that the explicitly marketing messages fall behind the individual user tweets indicates that they do not spread or influence the conversation as much as has been shown in previous years.

Marketing messages do spread when specific promotions are retweeted verbatim. A limited number of 140-character-or-less messages that focused on promotions and giveaways proliferated rapidly through retweets during the 100 days in this study. These retweets can be interpreted as public reactions [18], demonstrating that some attention toward promotion of regulations for e-cigarette prices and give-away promotions online is warranted. Taken together, however, explicit marketing content, although retweeted more, was a relatively small piece of the online conversation. While attention to policy and regulations for marketing content might be worthwhile, the more important public health effort is likely to focus on engaging in the conversation to create a more balanced perspective available to Twitter users.

### Limitations

As with all social media research and analyses, there are several limitations to this study. While an analysis that captured a wide breadth of tweets and retweets allowed for insights about large-scale theme and topics, this does not represent the exposure for all Twitter users. Individuals customize their Twitter experiences by following accounts, thus not all users would experience the content shared on social media in the same way. Additionally, although insights for individual user and proponent opinions can be made from content shared in the topics, this analysis does not reveal the impact of the tweets or retweets on perception and attitudes toward e-cigarettes from users who see this content. Lastly, no automated denoising technique was applied prior to text mining the tweets captured in this study; we took steps to reduce noise manually. As emerging techniques become more reliable [29], future studies should consider applying automated denoising techniques before analysis.

### Conclusions

As the prevalence of e-cigarettes continues to rise, it is important to know what messages about these products are potentially influencing consumer attitudes and use. This study is the first to uncover trending themes and topics from large-scale public conversations on social media.

While e-cigarette brands and distributors continue to use social media for e-cigarette marketing and promotion, these findings reveal that unique, large-scale consumer conversations are taking place on Twitter. Individuals are turning to social media to participate in discussions about policies, personal experiences, and the differentiation of e-cigarettes from traditional tobacco. Public health advocates should actively participate on social media to balance the conversation, and future research should investigate how these unique conversations influence attitudes toward and use of e-cigarettes.

## Abbreviations

API: application programming interface
e-cigarette: electronic cigarette
EM: expectation maximization
